# Modulation of metal-insulator transitions by field-controlled strain in NdNiO_3_/SrTiO_3_/PMN-PT (001) heterostructures

**DOI:** 10.1038/srep22228

**Published:** 2016-02-26

**Authors:** Seungyang Heo, Chadol Oh, Man Jin Eom, Jun Sung Kim, Jungho Ryu, Junwoo Son, Hyun Myung Jang

**Affiliations:** 1 Division of Advanced Materials Science (AMS) and Department of Materials Science and Engineering (MSE), Pohang University of Science and Technology (POSTECH), Pohang 790-784, Republic of Korea; 2 Department of Physics, Pohang University of Science and Technology (POSTECH), Pohang 790-784, Republic of Korea; 3 Functional Ceramics Group, Korea Institute of Materials Science (KIMS), Changwon 641-831, Republic of Korea

## Abstract

The band width control through external stress has been demonstrated as a useful knob to modulate metal-insulator transition (MIT) in *R*NiO_3_ as a prototype correlated materials. In particular, lattice mismatch strain using different substrates have been widely utilized to investigate the effect of strain on transition temperature so far but the results were inconsistent in the previous literatures. Here, we demonstrate dynamic modulation of MIT based on electric field-controlled pure strain in high-quality NdNiO_3_ (NNO) thin films utilizing converse-piezoelectric effect of (001)-cut 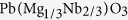
*-*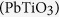 (PMN-PT) single crystal substrates. Despite the difficulty in the NNO growth on rough PMN-PT substrates, the structural quality of NNO thin films has been significantly improved by inserting SrTiO_3_ (STO) buffer layers. Interestingly, the MIT temperature in NNO is downward shifted by ~3.3 K in response of 0.25% in-plane compressive strain, which indicates less effective T_MI_ modulation of field-induced strain than substrate-induced strain. This study provides not only scientific insights on band-width control of correlated materials using pure strain but also potentials for energy-efficient electronic devices.

Correlated materials with partially occupied *d* electrons have aroused great interest due to extreme sensitivity of electronic phase transition with external stimuli^1^,^2^. Because of the sensitivity near the phase boundary, small changes in the crystal structures or charge density near a transition between competing phases can abruptly transform into different electronic phase, leading to large modulation in the electrical properties^3^,^4^,^5^. This sharp phase transition is expected to be advantageous for future electronic switching, requiring sharp on and off states in device application^6^,^7^. Therefore, the electronic devices exploiting Mott metal-insulator transition (MIT), i.e. ‘Mottronics’, may open up an important arena for fast and energy-efficient future electronics as a replacement of current logic switch^8^,^9^. Utilizing the transition in device application requires the capability to switch the distinct electronic phase with a control voltage in a reversible manner. To date, the control of electronic phase by gate bias has been demonstrated using the ionic electrolyte in most studies due to its facile carrier injection on the order of ~10^14^ cm^−2^, which sufficiently modulate the electronic state of correlated materials with large amount of carrier density^4^,^10^. However, electrolyte not only chemically alters the underlying correlated materials in some cases^11^,^12^, but its slow on/off switching time also limits the high-frequency performance for the practical applications. New ‘Mottronics’ device, so-called piezoelectronic transistor (PET), has been recently proposed, which modulates the channel conductance of strain-sensitive piezoresistive (PR) material by using a piezoelectric (PE) gate^7^. Simulations of the device performance using bulk materials properties show promising result for the future logic switch, predicting switching speed of ~4 GHz and lower power consumption. In this regard, strain-engineered phase transition in correlated materials becomes a core subject to investigate for electronic applications.

Among correlated materials, rare-earth nickelates (*R*NiO_3_, where *R* = trivalent rare-earth ion) exhibit a strain-sensitive MIT^13^,^14^,^15^,^16^,^17^: For example, their transition temperatures (T_MI_) have sensitively shifted in strained *R*NiO_3_, induced by either external hydrostatic pressure in bulk^14^,^15^ or biaxial stress imposed by a variety of substrates^16^,^17^,^18^,^19^,^20^,^21^. The origin of MIT modulation by external mechanical stress is attributed to the change in Ni-O bond lengths or the Ni-O-Ni bond angles, which leads to the modulation of band interactions via band-width control^13^,^14^. However, the influence of epitaxial strain on MIT characteristics has been inconsistent in several literatures, showing different T_MI_ under same lattice mismatch^16^,^17^,^19^,^20^,^21^,^22^. For instance, NdNiO_3_ (NNO) films grown on LaAlO_3_ substrates show different values of T_MI_, ranging from 175 K to 0 K, depending on growth condition^21^,^23^. To investigate pure strain effect on MIT, it is necessary to isolate strain effect from other stimuli and exclude the possibility to modulate defect concentration by different underlying substrates. Alternative approach to probe pure strain effect in the *R*NiO_3_ system is to take advantage of the converse piezoelectric effects in piezoelectric (or ferroelectric) substrates. By growing piezoresistive *R*NiO_3_ films on them, we are capable of *in-situ* manipulation of pure strain engineering of *R*NiO_3_. The most suitable candidate among piezoelectric substrates is 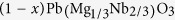
*-*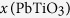 (PMN-PT), because PMN-PT single crystal with compositions near the morphotropic phase boundary (0.28 < *x* < 0.32) possesses excellent converse piezoelectric effects (*d*_*33*_ > 2000 pm/V)^24^,^25^ and thus provides the useful knob to dynamically control biaxial in-plane strain of *R*NiO_3_ thin films by gate voltage. This concept has been demonstrated in many magnetoelectric heterostructures, such as CoFe_2_O_4_/PMN-PT^26^, La_0.7_Sr_0.3_MnO_3_/PMN-PT^27^,^28^, La_0.8_Ca_0.2_MnO_3_/PMN-PT^29^ and SrRuO_3_/PMN-PT^30^, where the strain-mediated voltage control of magnetic properties is realized through lattice-spin coupling. However, a few reports have been recently studied on the tuning of the MIT in the correlated materials system, such as VO_2_ and Fe_3_O_4_, through lattice-charge coupling^31^,^32^,^33^. In addition, despite the possibility of pseudomorphic growth of perovskite *R*NiO_3_ films on PMN-PT, no report has been demonstrated on *in-situ* manipulation of MIT using *R*NiO_3_/PMN-PT epitaxial heterostructures by electric field control.

In this Article, we report strain-mediated field control of electrical transport in epitaxial NNO thin films utilizing converse-piezoelectric effect of PMN-PT substrates for the first time. In particular, the structural quality of NNO thin films is significantly improved by inserting SrTiO_3_ (STO) buffer layers between them. The biaxial strain in PMN-PT induced by converse piezoelectric coupling is appropriately transferred into NNO epitaxial layers, leading to the shift of metal-insulator transition temperature in NNO thin films. Moreover, strain-mediated resistivity modulation is dynamically observed by sweeping a gate bias across the piezoelectric substrates. Consequently, these results provide not only scientific insights on the pure strain effect of the *R*NiO_3_ system, but also show technological potentials in achieving energy-efficient ‘Mottronic’ devices.

## Results

**Figure 1:**
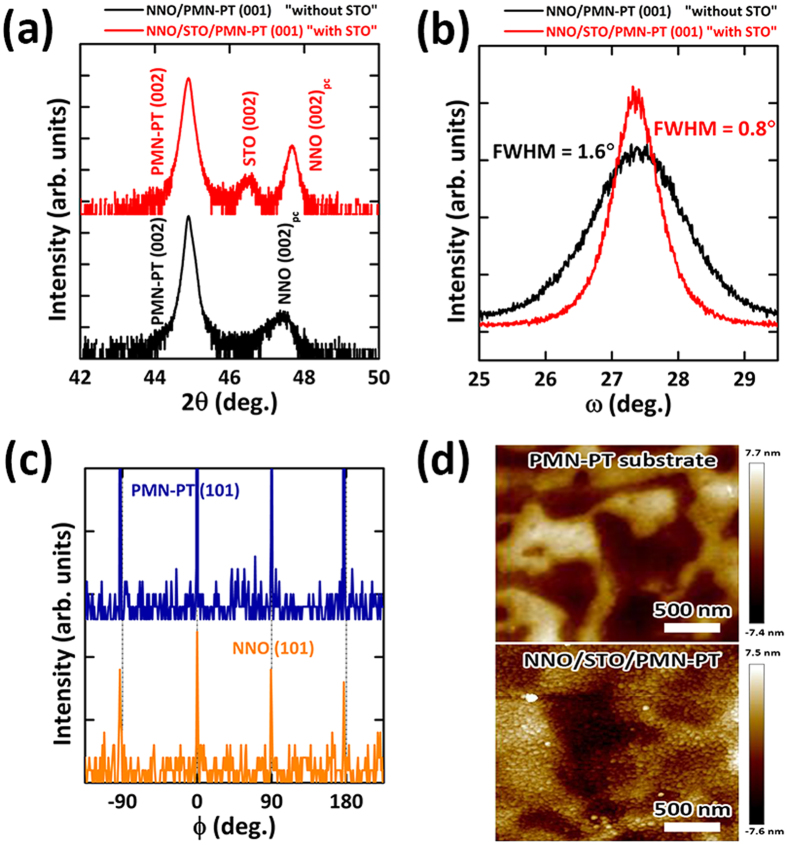
High-quality NNO epitaxial films grown on PMN-PT substrates by inserting STO buffer layer. (**a**) XRD θ-2θ scan of NNO/PMN-PT (001) (black solid line) and NNO/STO/PMN-PT (001) (red solid line). Note that NNO/STO/PMN-PT (001) shows higher and sharper (002) NNO peak than NNO/PMN-PT (001). (**b**) Rocking curves taken around NNO (002) diffraction for samples with and without STO buffer layer. The full width at half maximum (FWHM) of rocking curve around NNO (002) reflection with STO buffer layers is much smaller than that without STO buffer layers. (**c**) XRD phi (ϕ) scans taken on the NNO (101) and PMN-PT (101) diffraction peaks, respectively. (**d**) The surface morphology of the bare PMN-PT (001) substrate and NNO/STO/PMN-PT (001). The surface topography of NNO/STO/PMN-PT (001) is not degraded from that of the bare PMN-PT (001) substrate after the growth.

High-quality NdNiO_3_ (NNO) epitaxial thin films were grown on (001)-oriented PMN-PT single crystal substrate using SrTiO_3_ (STO) buffer layer by pulsed laser deposition. As an initial attempt, the direct growth of NNO thin films on PMN-PT substrates turned out to be very challenging, which leads to rough NNO films without MIT characteristics due to large lattice mismatch and relatively rough underlying substrates (Fig. S1, S2). Instead, our result clearly shows that the quality of epitaxial NNO thin films was significantly improved by inserting STO buffer layer. Figure 1(a) shows the comparison of XRD 2*θ*−*θ* scan of NNO (100 nm)/PMN-PT (001) (“without STO”, black solid line) and NNO (100 nm)/STO (25 nm)/PMN-PT (001) (“with STO”, red solid line). The observation of (002) NNO peak near (002) PMN-PT substrate peak indicates that the NNO films were highly c-oriented in both cases. Notably, the heterostructures “with STO” shows higher and sharper (002) NNO peak than the heterostructures “without STO”, representing better crystallinity of NNO by inserting STO buffer layers between NNO and PMN-PT substrate. In fact, as shown in Fig. 1(b), the full width at half maximum (FWHM) of rocking curve around NNO (002) reflection with STO buffer layers (0.8°) was much smaller than that without STO buffer layers (1.6°), which also confirms an improved crystallinity of NNO by STO buffer layer. In terms of peak position, the (002) peak of NNO with STO (47.67°) was closer to that of bulk NNO (002) peak (47.74°) than that of NNO without STO (47.35°). Considering the fact that the (002) peak tends to shift to the higher scattering angle with decreasing oxygen deficiency or moving toward cation stoichiometry in *R*NiO_3_ in the previous reports, this result provides the indirect evidence that point defects in NNO films are relatively suppressed with STO buffer layers^34^,^35^. Furthermore, in-plane epitaxial relationship between NNO thin films and PMN-PT substrate was analyzed by the XRD phi (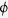) scan (Fig. 1(c)). The 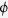
*-*scan spectrum of the NNO layers on (101) plane was characterized by a four-fold symmetry and matched well with that of the PMN-PT substrate, demonstrating that NNO thin films have an identical in-plane orientation with PMN-PT and undergo cube-on-cube epitaxial growth on the STO/PMN-PT (001). In addition to improved crystal quality through STO buffer layer, surface topography of NNO thin films on STO/PMN-PT (001) (RMS roughness (R_s_) ~ 2.49 nm, the bottom of the Fig. 1(d)) was not degraded from that of the bare PMN-PT (001) substrate (R_s_ ~ 2.35 nm, the top of the Fig. 1(d)) after the growth, which shows the improved surface topography compared to NNO thin films directly on PMN-PT substrate (R_s_ ~ 3.43 nm) (Fig. S1).

**Figure 2:**
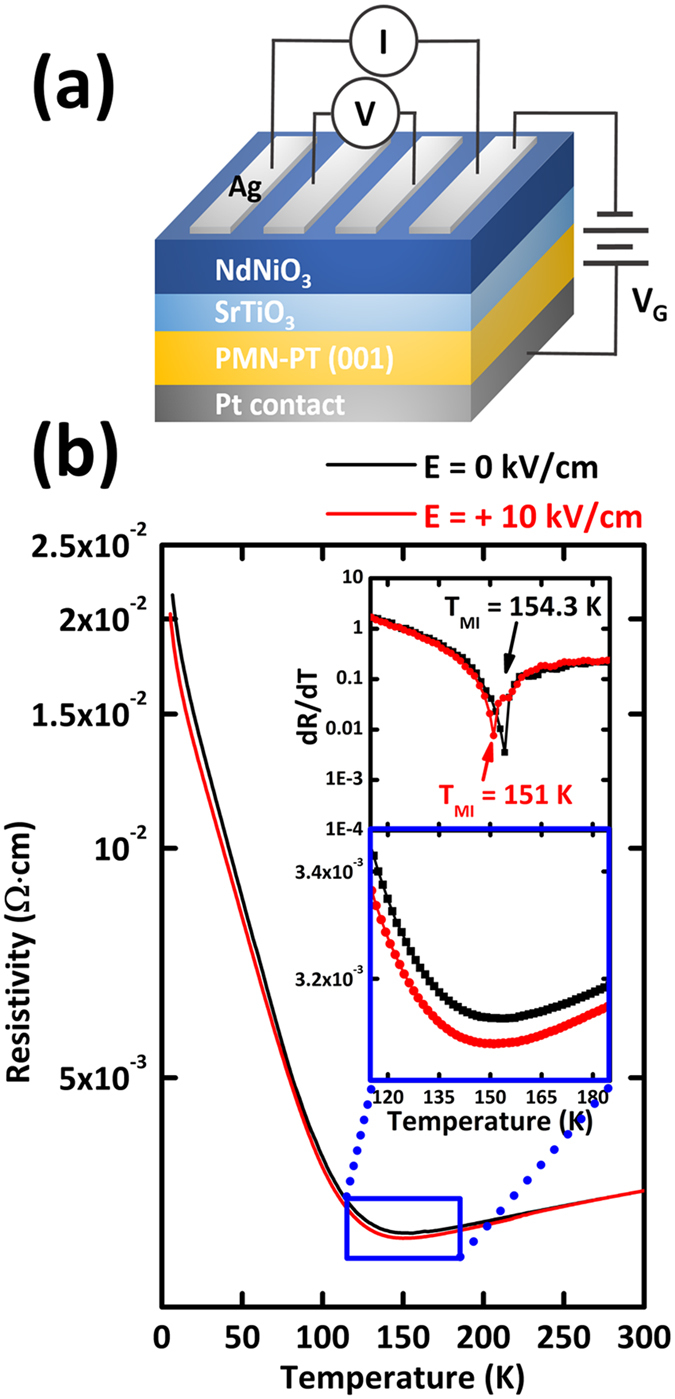
Modulation of metal-insulator transition of NNO by field-controlled piezo-strain of PMN-PT. (**a**) Schematics of four-point-probe resistivity measurement using voltage-induced strain of NNO/STO/PMN-PT (001) heterostructure. (**b**) Temperature dependence of the in-plane resistivity for the NNO thin films without out-of-plane electric field (black solid line) and with out-of-plane electric field of +10 kV/cm (red solid line). The insets shows the magnified plot of temperature dependence of resistivity and the first derivative of resistivity with respect to temperature (dR/dT) plot as a function of temperature between 120 K and 180 K.

In considering the results above, it is likely that STO buffer layers play a critical role on the improvement of NNO epitaxial films on PMN-PT substrate. Indeed, our initial growth of NNO thin films directly on PMN-PT (001) substrate yielded low-quality NNO thin films, presumably due to the following multiple origins: 1) the difficulty in synthesizing Ni^3+^ in NNO due to required high oxygen pressure in vacuum growth technique, such as pulsed laser deposition^36^, 2)intrinsically rough surface and inferior crystal quality with wide full width half maximum (Fig. S7) of PMN-PT substrate compared to other well-prepared perovskite substrates with unit cell step^37^, and, more importantly, 3) large lattice mismatch between NNO (a_pc_ = 0.3807 nm) and PMN-PT (a_pc_ = 0.4022 nm). Resulting from the low structural quality of epitaxial layer, no clear MIT was observed in the whole temperature range in NNO without STO buffer layer, representing inferior electrical transport properties (Fig. S2). On the other hands, due to the improved structural quality by STO buffer layer, our NNO/STO/PMN-PT heterostructures demonstrates clear MIT at 154.3 K as shown in Fig. 2(b), confirming the superior electrical property by inserting STO buffer layer. The transition temperature of 154.3 K is comparable to that of NNO films grown on STO substrates, pointing out that the “un-biased” in-plane lattice constant of NNO films is determined by that of relaxed STO buffer layer and thus lattice mismatch is likely to be accommodated through the STO buffer layer^37^. Therefore, the most probable origin of improvement by STO buffer layer is attributed to the effective reduction of large lattice mismatch (~5.65%) between NNO and PMN-PT. By inserting STO buffer layer with appropriate lattice parameter (a_c_ = 0.3905 nm), which is 3.0% less than that of PMN-PT and 2.5% greater than that of NNO, huge strain between NNO and PMN-PT enables to be gradually mitigated. The buffer layer allows for a better accommodation of lattice mismatch and thus gives rise to less defect formation in functional NNO layer. Moreover, Pb in PMN-PT is prone to incorporate into a functional film, i.e., NNO in our case, deposited on top of PMN-PT at high temperature growth process. STO buffer layers are likely to act as a diffusion barrier of volatile element Pb from the substrates by preventing from Pb inter-diffusion into NNO^37^.

Using our high-quality NNO films grown on PMN-PT with STO buffer layer, we explored field-controlled strain tuning of MIT in the epitaxial NNO thin films. To probe the field-induced strain effect of electrical transport on NNO (25 nm)/STO (10 nm)/PMN-PT heterostructure, out-of-plane electric field of +10 kV/cm was applied across the PMN-PT substrates at room temperature and subsequently the in-plane resistivity of NNO films was measured with four probe technique under cooling while out-of-plane electric field continued to be applied as described in Fig. 2(a). To ensure the direction of electric field, an electric bias was always applied to the Pt bottom electrodes and the top electrode was grounded. Here, we note that the voltage drop in STO film can be ignored due to the negligible thickness of STO films (10 nm) compared to PMN-PT substrates (0.2 mm). Moreover, the influence of STO buffer layer on in-plane resistivity can also be neglected because of much larger resistivity of STO compared to metallic NNO films. Figure 2(b) presents the temperature dependence of the in-plane resistivity for the NNO thin films without out-of-plane electric field (black solid line) and with out-of-plane electric field of +10 kV/cm (red solid line). Once the sample was poled with a DC electric field of +10 kV/cm, the resistivity decreased over all temperature range (red solid line). In particular, as the temperature approached to the T_MI_, resistivity modulation by electric field was greatly increased presumably due to the coexistence between metallic and insulating phase near T_MI_^5^,^31^,^32^. This can be more clearly seen in the first derivative of resistivity with respect to temperature (dR/dT) plot as a function of temperature (Inset of Fig. 2(b)), which demonstrates the reduction in the T_MI_ of ~3.3 K.

**Figure 3:**
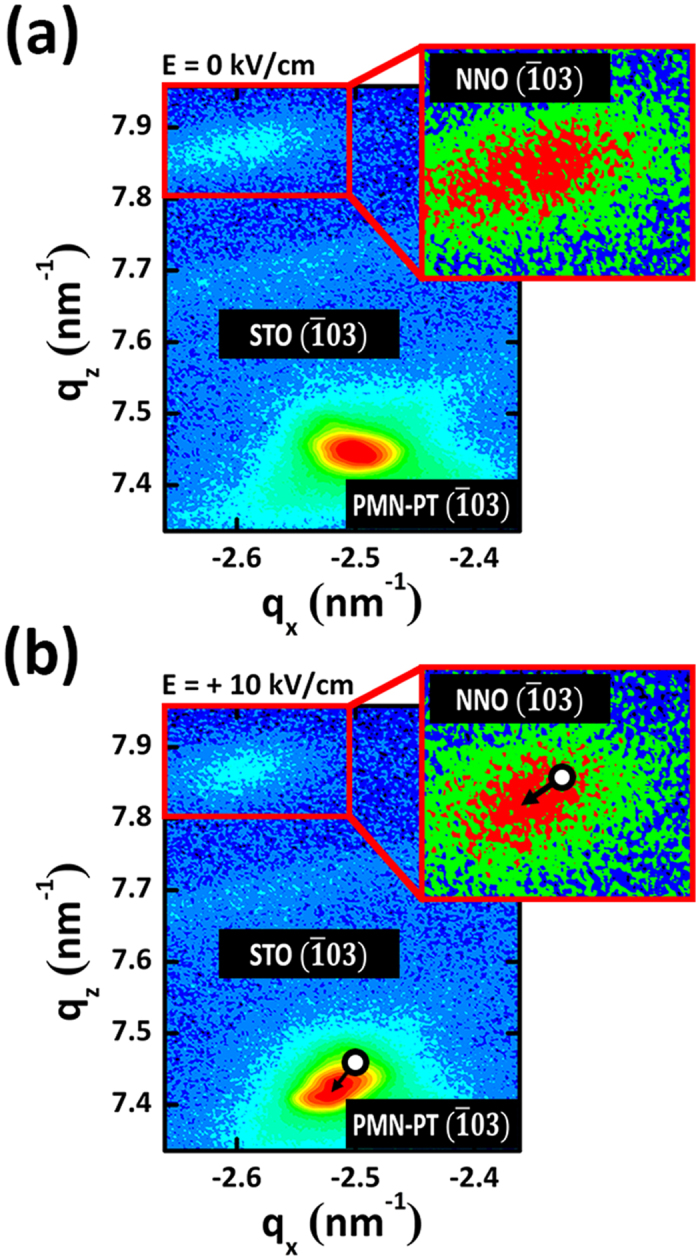
Structural modulation of NNO and PMN-PT by field-controlled piezo-strain. *in-situ* XRD reciprocal space mapping (RSM) around the (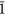
03) Bragg reflections of NNO/STO/PMN-PT heterostructure before (**a**) and after (**b**) the application of *in-situ* electric field of +10 kV/cm along [001] direction at room temperature (300 K). The insets presents the magnified RSM around (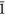
03) NNO reflection.

To elucidate the effectiveness of strain transfer to the NNO thin films by the field-induced strain more clearly, we carried out *in-situ* reciprocal space mapping (RSM) around the (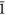
03) Bragg reflections of NNO (25 nm)/STO (10 nm)/PMN-PT heterostructure before (Fig. 3(a)) and after (Fig. 3(b)) the application of *in-situ* electric field of +10 kV/cm along [001] direction. Details of *in-situ* measurements are in Experimental Methods part and Supplementary Information (Fig. S5, S6). As shown in Fig. 3(a), substrate and film peaks exhibit different Q_x_ values, revealing that both STO buffer layers and NNO thin films are relaxed on the PMN-PT (001) substrate. The in-plane and out-of-plane lattice constant of “un-biased” NNO films were estimated to be 0.3861 nm and 0.3809 nm, respectively, which indicates that “un-biased” NNO films exhibits slightly in-plane tensile strain compared to bulk NNO. When the electric field of +10 kV/cm was applied to the PMN-PT along [001], the (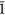
03) Bragg reflections of the PMN-PT substrate was shifted to the diagonal left direction (bottom black arrow), representing that out-of-plane lattice parameter increased and the in-plane lattice parameter shrunk through converse piezoelectric effect of PMN-PT. More importantly, the (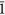
03) Bragg reflections of the NNO thin films was also shifted to the diagonal left direction (upper black arrow), which confirms the field-induced strain by converse piezoelectric effect in PMN-PT was appropriately transferred into NNO layer deposited on top. No impurity phase was induced before and after the application of the electric field (Fig. S4). The out-of-plane and in-plane lattice parameter of PMN-PT and NNO was modulated to be 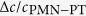 = +0.28%, 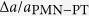 = −0.63% and 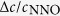 = +0.09%, 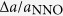 = −0.25%, estimated from 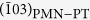 and 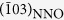 peak shift, respectively. For an epitaxial film under 2D stress, the Poisson ratio 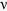 is given by 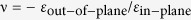
38: the out-of-plane strains (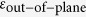) and in-plane strains (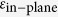) are equivalent to the modulation of out-of-plane (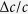) and in-plane lattice parameters (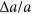), respectively. Based on this relationship, the Poisson ratio was estimated to be 0.44 and 0.35 for our PMN-PT substrates and NNO films, respectively. It should be noted that the Poisson ratios (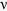) of the PMN-PT (001) and LaNiO_3_ have been reported to be 0.46^37^ and ~0.3^39^ in the previous literatures, respectively, confirming that our lattice parameter modulation results from mechanical response by piezoelectric strain in the each PMN-PT and NNO layer. Furthermore, there is negligible change of unit-cell volume (0.0568 nm^−3^ to 0.0566 nm^−3^) of NdNiO_3_ after the application of electric field (Table S3 in Supplementary Information), providing direct evidence that field-controlled piezoelectric strain does not influence the defect concentration and thus field-induced strain are able to modulate only band-width by the elastic coupling without changing the concentration of defects. On the other hand, the modulation of biaxial in-plane compressive strain in NNO (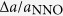 = −0.25%) was reduced compared to that in PMN-PT (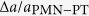 = −0.63%). This indicates that all piezoelectric strain of PMN-PT was not perfectly transferred into the NNO films. The inefficient strain transfer may be attributed into either the loss of strain energy in STO buffer layer or at the semi-coherent interface between STO and PMN-PT. Further investigation is needed to elucidate the origin of the loss of strain energy.

**Figure 4:**
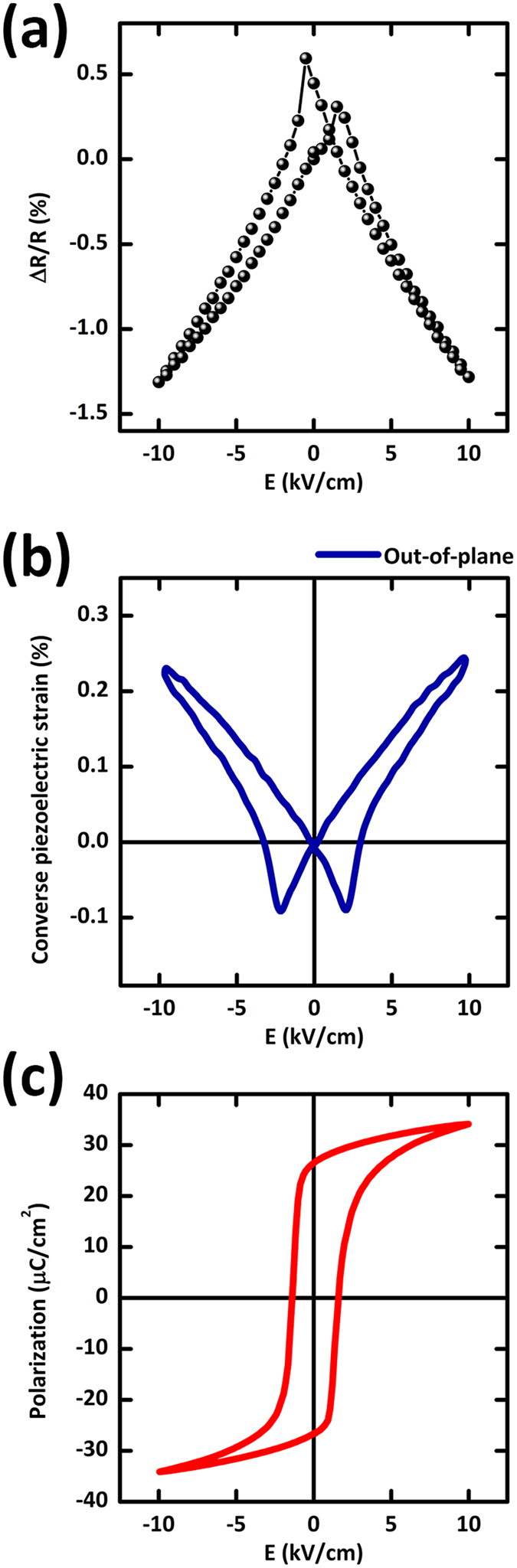
Relationship between *in-situ* relative change of in-plane resistivity in NNO and piezo-strain of PMN-PT. (**a**) The relative change of resistivity of the NNO thin films (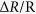) at 300 K as a function of bipolar electric field of ±10 kV/cm. (**b**) Out-of-plane piezo-strain versus E (S-E) for the PMN-PT substrate at 300 K. (**c**) The P-E hysteresis loop for the PMN-PT substrate at 300 K. The coercive field value (E_c_) is ±2 kV/cm at 300 K.

To clarify the effect of piezoelectric strain on the electrical transport in NNO thin films, we also measured *in-situ* relative change of resistivity in the NNO thin films (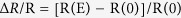, where 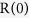 is the resistivity value without electric field) by sweeping a gate bias between ± 10 kV/cm at 300 K as shown in Fig. 4(a). After applying the electric field to the PMN-PT substrate from 0 V/cm to −10 kV/cm in the negative direction, it was swept from −10 kV/cm to +10 kV/cm and then switched back to 0 kV/cm. Upon cycling the electric field with +10 kV/cm, a ‘butterfly’ shape hysteresis appeared in 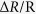 versus E plot, showing a relative resistivity change of ~1.5% at room temperature. This ‘butterfly’ shape is quite comparable to in-plane strain vs. E hysteresis loop of PMN-PT substrate, which can be calculated from measured out-of-plane strain vs. E hysteresis loop (Fig. 4(b)), but different from that of polarization vs. E hysteresis of PMN-PT substrate (Fig. 4(c)). This result provides the direct evidence that the butterfly-shaped resistivity modulation in NNO films is ascribed to the electric-field-induced piezo-strain, not to ferroelectric field effect (electrostatic doping) of the PMN-PT substrate. Considering infinitesimal screening length of electron in NNO films (1 ~ 2 nm)^6^, it is reasonable that the electrostatic doping effect by ferroelectric field can be ruled out in our 25 nm-thick NNO films. Instead, by sweeping the bias across the PMN-PT substrate, field-induced in-plane tensile (or compressive) strain was transferred to the epitaxial NNO layer, thereby giving rise to the increase (or decrease) of in-plane resistivity in NNO layers.

## Discussion

In the previous theoretical and experimental investigation, it is well established that compressive strain in crystal lattice induces the widening of the *d* bands of *R*NiO_3_, giving rise to both increase of conductivity and lowering of T_MI_^14^,^17^,^18^. In particular, external stress decreases (or increases) the Ni-O-Ni bond angle, narrowing (or widening) the band, and thus leading to the modulation of T_MI_^13^,^18^. In our case, by precisely controlling the lattice strain states by the electric field-induced compressive strain from piezoelectric substrate, the Ni-O-Ni bond angles, which are determined by the rotation of the oxygen octahedral, would change the overlap between the Ni-*3d* orbitals and O-*2p* orbitals which, in turn, leads to the decrease of resistivity and the shift in the T_MI_. Despite the improved NNO quality by using STO buffer layer, the loss of strain transfer in our heterostructure still inhibits better resistivity modulation of NNO layer by gate voltage. Therefore, better electrical tuning of resistivity in NNO, i.e. greater resistivity modulation and lower turn-on voltage, could be achieved after the optimization of the heterostructure for improved strain transfer.

**Figure 5:**
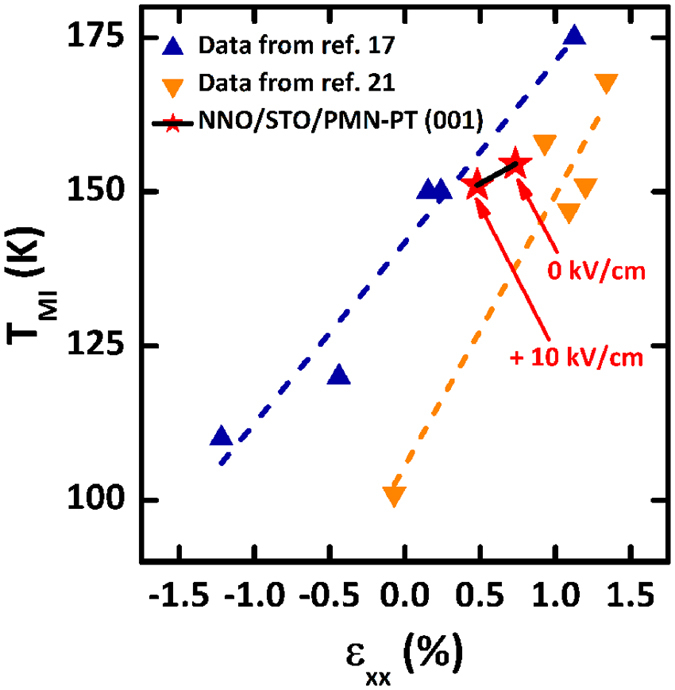
Comparison of the T_MI_ modulation of NNO thin films as a function of field-controlled strain (this work) with that as a function of substrate-controlled strain (previous works). Data for T_MI_ as a function of substrate-controlled in-plane strain (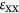) were chosen from ref. 17 (blue) and ref. 21 (orange). Two red asterisks indicate the T_MI_ modulation as a function of field-controlled in-plane strain (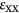) in our NNO thin films grown on STO/PMN-PT (001) substrate.

Unlike typical method for substrate-controlled strain engineering using lattice mismatch, our method using converse piezoelectric effect enables to dynamically control the strain transferred to the NNO layer as a function of gate voltage. Moreover, by using field-controlled strain engineering in our heterostructure, we are capable of separating pure strain effect on the modulation of MIT from influence of other stimuli, e.g. different defect concentration, induced by different substrates. To quantitatively compare the T_MI_ modulation of field-controlled strain in our work with that of substrate-controlled strain in previous works^17^,^21^, we plot a systematic relationship between in-plane strain (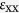) and T_MI_ of NNO film, as shown in Fig. 5. To accurately obtain the in-plane film strain from our data and the previous literatures, the unstrained film lattice parameter (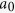) is taken by


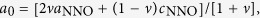


where 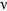, 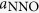 and 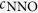 indicates the Poisson’s ratio, in-plane and out-of-plane lattice parameter of NNO, respectively. From this relationship, the unstrained lattice parameter (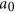) of the “un-biased” NNO films was calculated to be ~0.3833 nm, which is greater than that of the bulk pseudocubic NNO (0.3807 nm) but comparable to other high-quality NNO epitaxial layer grown on well-prepared perovskite substrates^21^. The in-plane film strain (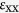) was calculated as 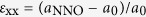, using the unstrained film lattice parameters determined as described above, confirming the strain state of “un-biased” NNO films as a +0.73% tensile strain on STO/PMN-PT. The detailed calculation procedure of in-plane film strain is described in Supplementary Information (Fig. S3, Table S1, Table S2). Interestingly, while substrate-controlled strain modulates the transition temperature of ~7.35 K and ~10.97 K per 0.25% in-plane strain in the previous reports, our field-controlled strain shifts the temperature of only ~3.3 K per 0.25% strain. This result indicates that field-controlled strain appears to be less effective to modulate the metal-insulator transition in NNO films compared to substrate-controlled strain. This discrepancy may be related to the fact that substrate-controlled strain is likely to simultaneously modulate the defect concentration, as well as the rotation of the oxygen octahedral for band-width control in the NNO films on top. In the previous report, it is claimed that oxygen vacancies provide a channel for relieving tensile strain in complex oxides grown on substrates with large lattice parameters and thus more oxygen vacancies can be created for strain accommodation in tensile-strained films^40^,^41^. In particular, Ni^3+^valence state is less stabilized by a tensile strain and tensile strain induces the formation of oxygen vacancies in *R*NiO_3_^40^. In fact, previous work using lattice mismatch suffered from inconsistencies to tune MIT transitions of NNO films as shown in Fig. 5. The inconsistent T_MI_ even under same substrate may be explained by divergent oxygen contents in the NNO films, which strongly suggests that the influence of defects cannot be ruled out in case of strain engineering using different substrate. Therefore, substrate-induced strain appears to overestimate the strain-induced modulation of T_MI_. On the other hand, field-induced strain are able to modulate only pure strain without changing the concentration of defects and, therefore, our approach is suitable for studying pure strain effect on transport property of strongly correlated oxide materials.

## Conclusion

In summary, we successfully grew high-quality NdNiO_3_ (NNO) thin films on PMN-PT (001) substrate by inserting SrTiO_3_ (STO) buffer layers between them. In particular, the crystal quality and electrical transport of NNO thin films has been significantly improved due to the reduction of the lattice mismatch with PMN-PT. Using NNO/STO/PMN-PT heterostructure, the electrical transport properties in NNO layer was modulated by the field-controlled piezoelectric strains, which is induced and transferred by converse piezoelectric effect of PMN-PT single crystal substrates. Interestingly, the metal-insulator transition temperature in NNO was shifted by ~3.3 K in response of 0.25% in-plane compressive strain, which indicates less effective T_MI_ modulation of field-induced strain than substrate-induced strain. These results provide not only scientific insights on band-width control of correlated materials using pure strain but also opportunities for novel energy-efficient electronic devices.

## Experimental Methods

Epitaxial NdNiO_3_ (NNO) thin films and SrTiO_3_ (STO) buffer layers were grown on one-side-polished (001)-oriented PMN-PT single-crystal substrates (CPSC160-95, 0.2 mm thickness, Ceracomp, South Korea) without breaking the vacuum using pulsed laser deposition with a KrF excimer laser fluence of ~1.5 J/cm^2^ (wavelength 248 nm; Coherent, Compex Pro 102 F). The growth of STO buffer layer was carried out at 720 °C in an O_2_ partial pressure of 100 mTorr, while NNO layers were grown at 550 °C in an O_2_ partial pressure of 190 mTorr. After growth, the sample was exposed immediately to a higher pressure 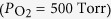 for *in-situ* post-annealing at growth temperature (550 °C) for 30 min to minimize the oxygen deficiency. The surface morphology of films was obtained using an atomic force microscope (AFM, VEECO Dimension 3100). The crystallinity and thickness of the films were characterized with a high-resolution x-ray diffraction (XRD) and x-ray reflection (XRR) measurement (Bruker D8 Discover X-ray diffractometer). The *in-situ* reciprocal space mapping (RSM) was also carried out by using a Bruker D8 Discover X-ray diffractometer. In order to align NdNiO_3_ film peak, PMN-PT substrate peak was used as a reference. All *in-situ* RSM measurements (0 kV/cm and +10 kV/cm) were performed at room temperature (300 K) without changing the temperature. In order to apply electric field during measurement, Pt bottom electrodes were deposited on the backside of the PMN-PT substrate by a magnetron dc sputtering system, while the conducting NNO layer acts as top electrode. Keithley-6517B electrometer was utilized to provide DC electric field up to +10 kV/cm. More information about RSM measurements are in Supplementary Information (Fig. S5, S6). The in-plane resistivity was measured using the standard four-point-probe method in a Physical Property Measurement System (PPMS, Quantum Design, Fig. 2(a)). Gate sweep resistivity of the sample were measured by Van der Pauw method with DC electric fields of up to 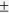 10 kV/cm, provided by a Keithley-6517B electrometer and were applied between the top NNO film and the bottom Pt electrode on the underside of PMN-PT (001) substrate. The converse piezoelectric behavior (S-E hysteresis) and the P-E hysteresis curves of PMN-PT single crystal substrate were measured by a Laser Displacement Sensor (LK-G10, Keyence Co. Tokyo, Japan, 10 nm resolution with a spot size of 20 μm) and a ferroelectric test system (P-LC100-K, Radiant Technologies, Albuquerque, NM) at 10 Hz, respectively.

## Additional Information

**How to cite this article**: Heo, S. *et al.* Modulation of metal-insulator transitions by field-controlled strain in NdNiO_3_/SrTiO_3_/PMN-PT (001) heterostructures. *Sci. Rep.* **6**, 22228; doi: 10.1038/srep22228 (2016).

## Supplementary Material

Supplementary Information
