# Supplementary material for: Modulation of metal-insulator transitions by field-controlled strain in NdNiO3/SrTiO3/PMN-PT (001) heterostructures
Source: Sci Rep. 2016 Feb 26;6:22228. doi: 10.1038/srep22228 (PMC4768092; doi:10.1038/srep22228)
Supplement: Supplementary Information [file srep22228-s1.pdf]

Supplementary information for

Modulation of metal-insulator transitions by field-controlled strain in $NdNiO_3/SrTiO_3$/PMN-PT (001) heterostructures

Seungyang Heo[1)], Chadol Oh[1)], Man Jin Eom[2)], Jun Sung Kim[2)], Jungho Ryu[3)], Junwoo Son[1),*], and Hyun Myung Jang[1),*]

[1)] Division of Advanced Materials Science (AMS) and Department of Materials Science and Engineering (MSE), Pohang University of Science and Technology (POSTECH), Pohang 790-784, Republic of Korea

[2)] Department of Physics, Pohang University of Science and Technology (POSTECH), Pohang 790-784, Republic of Korea

[3)] Functional Ceramics Group, and Advanced Characterization & Analysis Group, Korea Institute of Materials Science (KIMS), Changwon 641-831, Republic of Korea

* jwson@postech.ac.kr, hmjang@postech.ac.kr

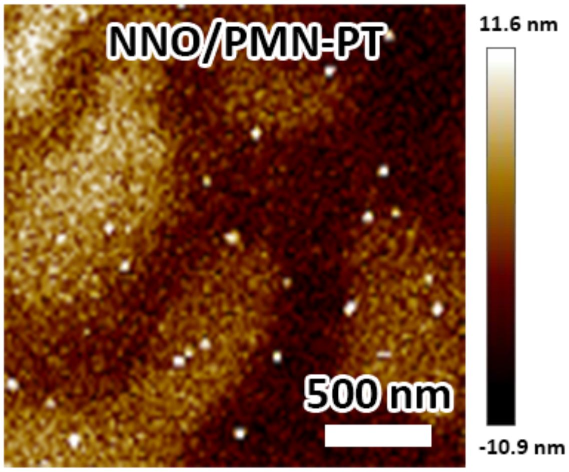

Figure S1 | AFM images of NNO thin films directly on PMN-PT (001) substrate. RMS roughness (R_s) of the NNO/PMN-PT is about 3.43 nm, which is rougher than the NNO/STO/PMN-PT (R_s ~ 2.49 nm).

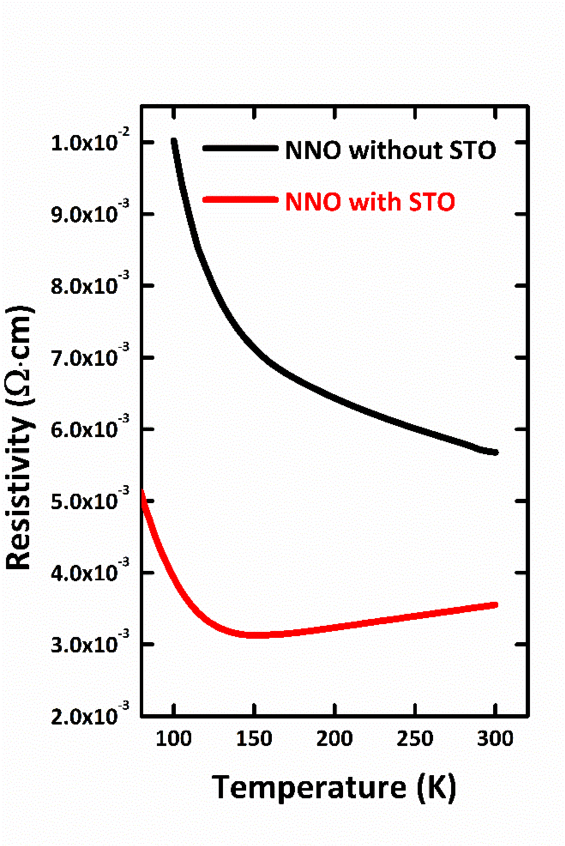

Figure S2 | Temperature dependence of the resistivity for the NNO thin films without STO (black solid line) and with STO (red solid line). The room temperature resistivity of $NdNiO_3$ is usually good indicator of the degree of disorder, e.g. degree of oxygen deficiency; the more oxygen deficiencies in nickelate systems, the higher room temperature resistivity and stronger carrier localization by disorder. The strong carrier localization in highly resistive films usually prevents the observation of metal-insulator transition. Unlike NNO/STO/PMN-PT, obviously, NNO/PMN-PT (NNO directly on PMN-PT) shows higher room temperature resistivity and no distinct metal-insulator transition, indicating high degree of disorder and strong carrier localization. Therefore, NNO grown on STO buffer show distinct metal-insulator transition, as well as lower room temperature resistivity, which indicates that carrier localization by defects is suppressed in NNO/STO/PMN-PT by using STO buffer layer.

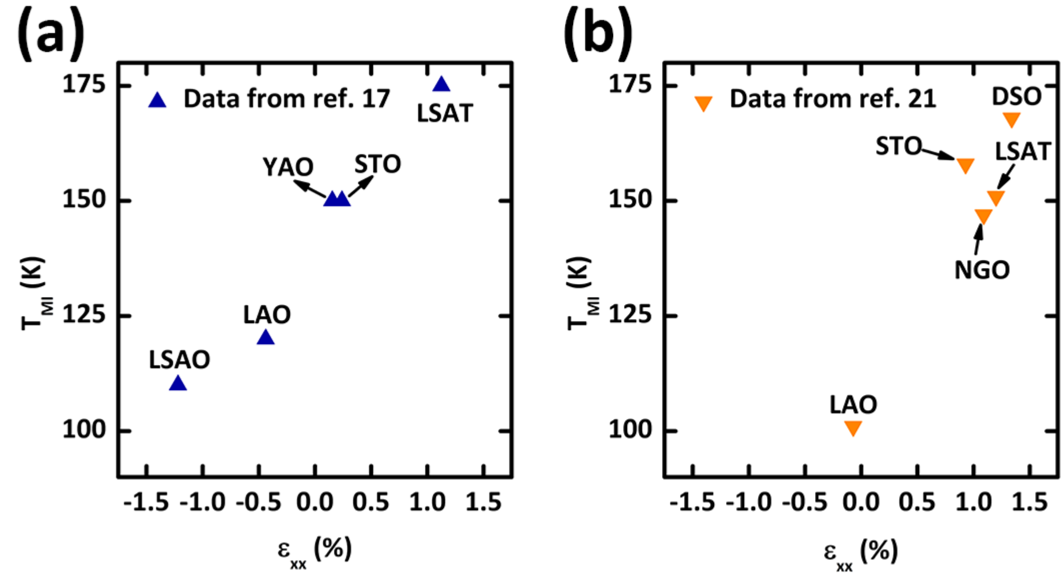

Figure S3 | T_{MI} as a function of ε_{xx} for NNO thin films on various substrates in (a) ref. 17 (blue) and (b) ref. 21 (orange).

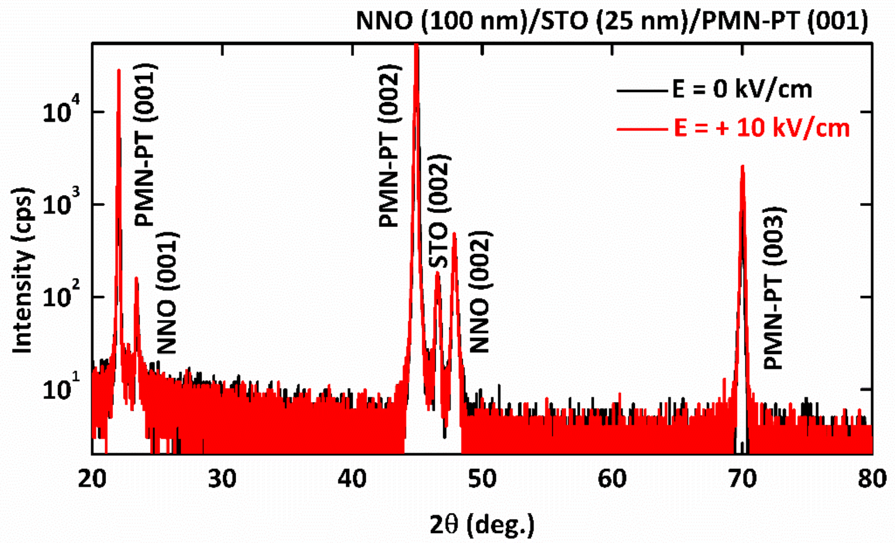

Figure S4 | Wide angle XRD patterns of NNO/STO/PMN-PT (001) at different bias conditions of 0 kV/cm and + 10 kV/cm. No impurity phases (such as NiO) are observed within the detection level of XRD.

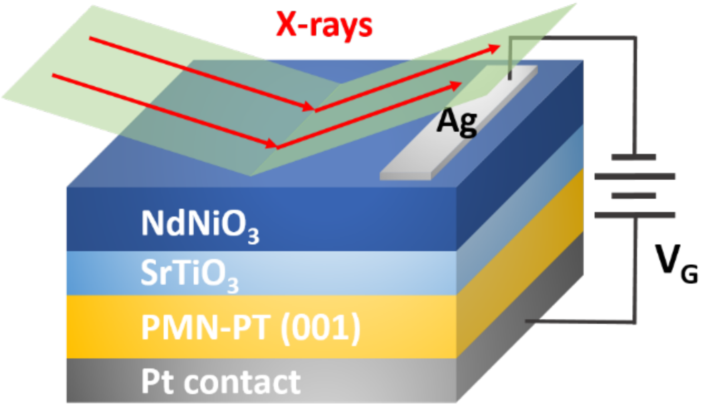

Figure S5 | Schematic of the experimental set-up for RSM measurements using X-ray diffractometer when an electric field is applied to the PMN-PT along the thickness direction. In order to align $NdNiO_3$ film peak, PMN-PT substrate peak was used as a reference. All in-situ RSM measurements (0 kV/cm and + 10 kV/cm) were performed at room temperature (300 K) without changing the temperature.

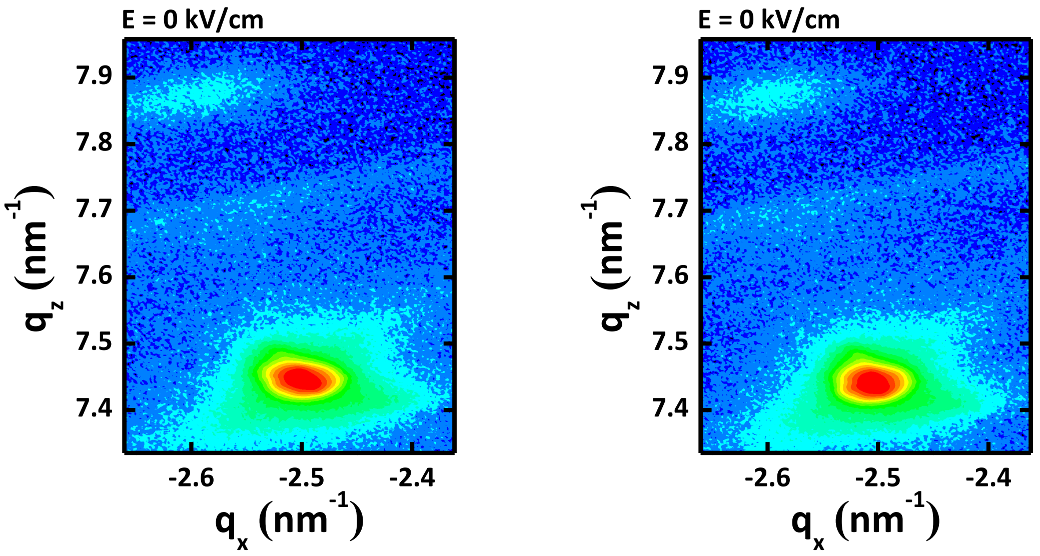

Figure S6 | (Left) RSM data of initial measurement (same as Fig. 3(a)). (Right) RSM data after electric field is removed. RSM data were collected in the sequence as follow, to see reproducibility of the data point: (1) 0 kV/cm → (2) + 10 kV/cm → (3) 0 kV/cm. Both of (1) and (2) are presented in the Fig. 3. The RSM of the sequence (3), i.e. after electric field is removed, is presented in the right of the Fig. S6, showing that both PMN-PT substrate peak and NNO peak are recovered to the original position reversibly, clearly showing reversible structural responses. Therefore, we can rule out the irreversible structural response as the origin of the reduced response of temperature shift with strain. In addition, the error in the RSM data could be neglected and the RSM data can be reproducible.

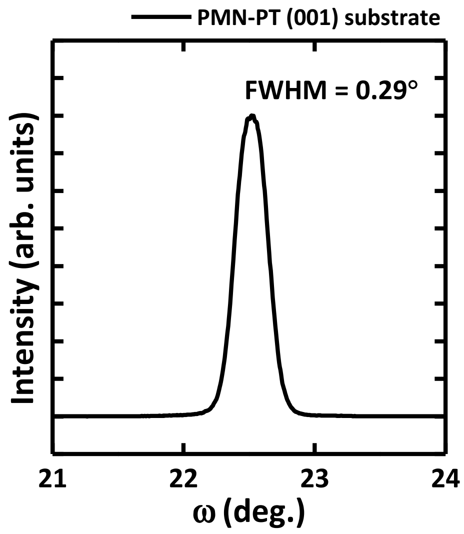

Figure S7 | Rocking curves taken around PMN-PT (002) diffraction. All PMN-PT substrates usually have intrinsically inferior crystal quality, such as large FWHM value and rough surface, compared to other well-prepared perovskite substrates (e.g. $SrTiO_3$ substrates with FWHM < 0.05°). Despite the fact that our optimized $NdNiO_3$ films shows relatively large FWHM (~ 0.8°) in Fig. 1(b) due to intrinsically inferior crystal quality of PMN-PT substrates, the crystal quality of our $NdNiO_3$ epitaxial layers on PMN-PT was significantly improved by inserting $SrTiO_3$ buffer layers.

Ref. 17	a_{NNO} (nm) (in-plane)	c_{NNO} (nm) (out-of-plane)	a_0 (nm) (unstrained)	ε_{xx} (%)	T_{MI} (K)
NNO on LSAT	0.3868	0.3788	0.3825	+ 1.13	175
NNO on STO	0.3821	0.3804	0.3812	+ 0.24	150
NNO on YAO	0.3821	0.381	0.3815	+ 0.16	150
NNO on LAO	0.379	0.3821	0.3807	– 0.44	120
NNO on LSAO	0.3754	0.384	0.38	– 1.22	110

Table S1 | Lattice parameters, ε_{xx}, and T_{MI} of NNO thin film on various substrates in ref. 17. The unstrained lattice parameters (a_0) are calculated from the equation,

$$a_0 = [2\nu a_{\mathrm{NNO}} + (1-\nu)c_{\mathrm{NNO}}]/[1+\nu],$$

where ν, a_{NNO} and c_{NNO} indicates the Poisson's ratio, in-plane and out-of-plane lattice parameter of NNO, respectively. The in-plane film strain (ε_{xx}) was calculated as $\varepsilon_{xx} = (a_{\mathrm{NNO}} - a_0)/a_0$, using the unstrained film lattice parameter determined as described above. The Poisson's ratio is set to 0.3 here for direct comparison with ref. 21.

Ref. 21 (300 mTorr)	ε_{xx} (%)	T_{MI} (K)
NNO on DSO	+ 1.34	168
NNO on STO	+ 0.93	158
NNO on LSAT	+ 1.2	151
NNO on NGO	+ 1.09	147
NNO on LAO	− 0.07	101

Table S2 | ε_{xx} and T_{MI} of NNO thin film on various substrates in ref. 21.

	a_{NNO} (nm) (in-plane)	c_{NNO} (nm) (out-of-plane)	Unit cell Volume (nm^{-3})
As-grown NNO (0 kV/cm)	0.3861	0.3809	0.0568
As-grown NNO (+ 10 kV/cm)	0.3852	0.3812	0.0566
Bulk NNO (Pseudocubic)	0.3804	0.3804	0.0550

Table S3 | Lattice parameters and unit cell volume of our $NdNiO_3$ films (on STO/PMN-PT (001)) before (0 kV/cm) and after the application of electric field (+ 10 kV/cm) along [001] direction, which was estimated from the in-plane and out-of-plane lattice constant from XRD RSM data (Fig. 3), and that of bulk $NdNiO_3$. Compared to stoichiometric bulk $NdNiO_3$, 2.9 % increase of unit-cell volume of our as-grown $NdNiO_3$ films is observed, which represents that our $NdNiO_3$ films still contains more defects, e.g. oxygen vacancies, than bulk $NdNiO_3$, despite significant improvement of crystal quality by inserting $SrTiO_3$ buffer layer. However, it should be noticed that *there is negligible change of unit-cell volume (0.0568 nm^{-3} to 0.0566 nm^{-3}) of $NdNiO_3$ after the application of electric field*. This provide direct evidence that field-controlled piezoelectric strain does not influence the defect concentration and thus field-induced strain are able to modulate only band-width without changing the concentration of defects.

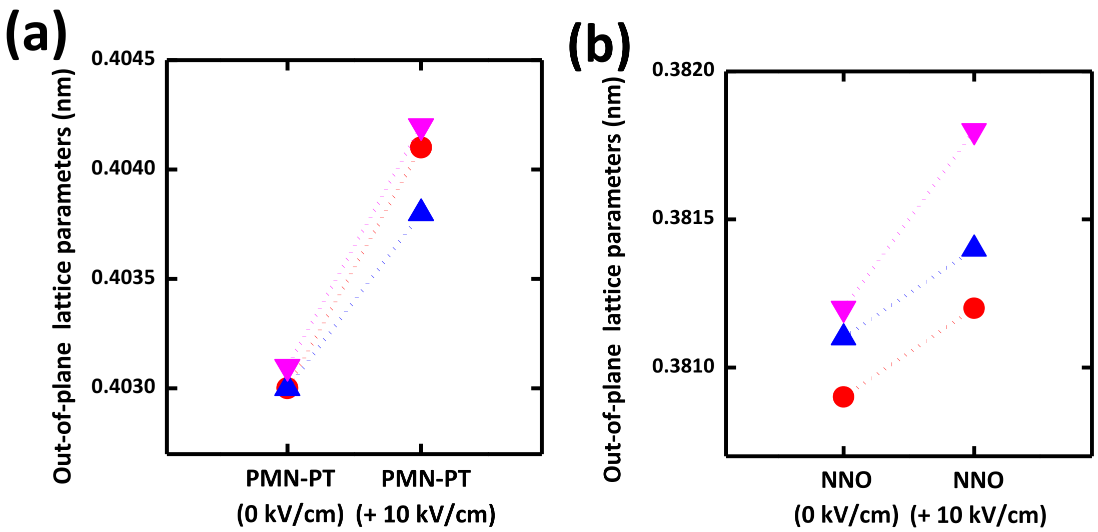

Figure S8 | Out-of-plane lattice parameters in repetitive measurements. The data set denoted as red solid circle indicates out-of-plane lattice constants of PMN-PT and NNO at each bias condition (0 kV/cm and + 10 kV/cm) extracted from the *in-situ* RSM measurement (Fig. 3). The out-of-plane lattice constant of blue and magenta symbol are extracted from repetitive XRD θ-2θ measurements of our NNO/STO/PMN-PT. Note that all out-of-plane lattice parameters of PMN-PT and NNO clearly increase when applying the electric field of + 10 kV/cm in all repetitive measurements, and it matches well with the out-of-plane lattice parameter changes in RSM measurement.
